# The impact of emotion on numerosity estimation

**DOI:** 10.3389/fpsyg.2013.00521

**Published:** 2013-08-09

**Authors:** Joseph M. Baker, Katrina S. Rodzon, Kerry Jordan

**Affiliations:** Department of Psychology, Utah State University, 2810 Old Main HillLogan, UT, USA

**Keywords:** Weber’s Law, numerical cognition, emotion, quantitative discrimination, quantity

## Abstract

Both time and numerosity can be represented continuously as analog properties whose discrimination conforms to Weber’s Law, suggesting that the two properties may be represented similarly. Recent research suggests that the representation of time is influenced by the presence of emotional stimuli. If time and numerosity share a common cognitive representation, it follows that a similar relationship may exist between emotional stimuli and the representation of numerosity. Here, we provide evidence that emotional stimuli significantly affect humans’ estimation of visual numerosity. During a numerical bisection task, enumeration of emotional stimuli (angry faces) was more accurate compared to enumeration of neutrally valenced stimuli (neutral faces), demonstrating that emotional stimuli affect humans’ visual representation of numerosity as previously demonstrated for time. These results inform and broaden our understanding of the effect of negative emotional stimuli on psychophysical discriminations of quantity.

## INTRODUCTION

A large body of work has documented similarities in representing numerical and temporal properties of stimuli (e.g., Meck and Church, 1983; Fetterman, 1993; Roberts et al., 2000; Bueti and Walsh, 2009). For example, discriminations of time and numerosity in human and non-human animals conform to Weber’s Law (e.g., Moyer and Landauer, 1967; Dehaene et al., 1998; Brannon and Roitman, 2003; Brannon et al., 2007; Jordan et al., 2008a,b; Jordan and Baker, 2011), indicating that both can be represented internally as continuous scalar properties. Both human and non-human animals simultaneously encode time and numerosity and are capable of distinguishing changes in either property when the other is held constant (Meck and Church, 1983; Brown, 1997; Brannon and Roitman, 2003; Walsh, 2003; Roitman et al., 2007; Lourenco and Longo, 2010). Neuroimaging and neuropsychological studies in humans provide further evidence for similar representation of numerosity and time. For instance, similar parietal activation is seen when processing time and numerosity (Rao et al., 2001; Pinel et al., 2004; Buhusi and Meck, 2005; Cantlon et al., 2006). However, while the above evidence suggests that a common mechanism may underlie representations of both numerosity and time, other evidence exists which implicates numerosity-specific cognitive mechanisms (e.g., Cohen Kadosh et al., 2008; Dormal et al., 2008, 2012).

The current study will help shed light on this debate by probing for convergent or divergent effects of time and number processing in response to a common stimulus property manipulation. For example, a growing body of research has begun to investigate the impact of emotion on temporal processing. In some temporal tasks, the duration of emotional events is significantly overestimated compared to the duration of neutral events (e.g., Droit-Volet et al., 2004; Gil and Droit-Volet, 2011). Furthermore, Gil and Droit-Volet (2011) recently found that visual stimulus duration estimation is more accurate for timing of angry (i.e., emotional) compared to neutral faces. Cocenas-Silva et al. (2012) also discovered that temporal memory for event durations was more accurate when learned in emotional compared to neutral conditions, and that learning temporal durations in threatening emotional conditions led to better temporal memory than learning durations in non-threatening emotional conditions.

Despite the increasing body of research investigating the effect of emotion on temporal perception, a distinct gap in our knowledge of emotion and representation of general quantity remains. Specifically, it remains unresolved whether emotion biases representation of other quantities, such as numerosity, similar to the manner in which it affects processing of time. The goal of the current research is thus to extend previous questions regarding emotion and temporal perception to the domain of numerosity.

### EFFECTS OF EMOTION ON TEMPORAL PROCESSING

Emotions play a critical role in human functioning and have been considered by some as the primary motivational system in humans, involved in organizing cognition, perception, and action (e.g., Tomkins and Karon, 1962). Emotions are considered to serve specific functions, including those related to behavioral, social, and internal regulations. Because of the important role emotions are hypothesized to serve in successfully navigating our physical and social environments, research has addressed how emotions affect perception; for example, how does emotion affect our perception of time? When experiencing stressful events, such as foot shocks (Meck, 1983) and forced eye contact with an angry face (Schiff and Thayer, 1970), results consistently support differing temporal estimations of emotional events compared to neutral events. For example, the duration of emotional events (Meck, 1983; Stetson et al., 2007) and emotional stimuli (Droit-Volet et al., 2004; Gil et al., 2007; Tipples, 2008) are often overestimated when compared to neutral stimuli.

Most relevant for the current research are experiments by Droit-Volet et al. (2004), Droit-Volet and Meck (2007), and Tipples (2008), in which researchers investigated how emotional stimuli impact visual temporal perception in a duration bisection task. In each of these studies participants were first trained to discriminate between two anchor durations (short vs. long), and were then required to classify intermediate durations as being more similar to the short or long anchor durations. Participants consistently judged the duration of the visual presentation of emotionally valenced faces (i.e., angry, happy, sad, or fearful) to be longer than the duration of neutral faces. Moreover, Gil and Droit-Volet (2011) report similar overestimations of emotional stimuli within duration bisection, verbal estimation, and production tasks, but not in temporal generalization or reproduction tasks. In fact, when asked to reproduce the learned duration of an exposure to angry and neutral faces, Gil and Droit-Volet (2011) report increased accuracy for timing of angry compared to neutral faces, suggesting that temporal overestimation of angry stimuli may be associated with relatively more accurate representations of angry compared to neutral stimuli in memory. Furthermore, this effect of emotion on time is not limited to the visual modality but has been investigated in the auditory modality as well (e.g., Angrilli et al., 1997; Noulhiane et al., 2007; Mella et al., 2011).

### EVIDENCE FOR A COMMON REPRESENTATION OF NUMEROSITY AND TIME

According to Weber’s law, discrimination of disparate magnitudes of a continuous property (e.g., brightness, size, time, numerosity, etc.) is mediated by the ratio between the magnitudes of the competing stimuli (Stevens, 1957). As this ratio approaches 1:1, discrimination becomes increasingly difficult. Conversely, as the ratio decreases, discrimination becomes easier. This ratio dependence suggests a “noisy” scalar representation, such that mental representations of similar magnitudes “overlap” and discrimination suffers. As the magnitudes become less similar, there is less mental overlap and discrimination improves. Adherence to predictions made by Weber’s Law in both human and non-human animals suggests similar representations of each adherent quantity across species (e.g., Walsh, 2003; Cantlon et al., 2006; Baker et al., 2011, 2012).

Evidence that time and numerosity may be represented by the same mechanism is found in various animal species (e.g., Meck and Church, 1983; Fetterman, 1993; Roberts et al., 2000). For example, Meck and Church (1983) showed that rats encode both time and numerosity simultaneously when the two dimensions are intentionally confounded. After training rats in a duration bisection procedure to make one response to a 2-s/2-cycle auditory stimulus and a different response to an 8-s/8-cycle auditory stimulus, researchers pitted time and numerosity against each other by varying one property while holding the other constant. Rats were equally accurate at discrimination of 4:1 ratios of both time and numerosity despite the confounding of these properties during training, suggesting that rats simultaneously encoded both properties. Furthermore, the point of subjective equality (PSE) – the quantity at which the rats were equally likely to categorize the stimulus as long or short (if tested on duration) or many or few (if tested on numerosity) – was equivalent for time and numerosity. Moreover, the administration of methamphetamine altered rats’ discrimination of time and numerosity similarly (Meck and Church, 1983).

Parallel work in humans shows similar behavioral and neural representation of time and numerosity (e.g., Roitman et al., 2007; Allman et al., 2012). These studies demonstrate that adult humans simultaneously represent time and numerosity and that these properties interact, suggesting a common mental magnitude coding for both. For example, infants’ discriminations of numerosity increase similarly in precision over development as their discriminations of temporal stimuli (e.g., Brannon et al., 2007). Using a visual habituation paradigm, Brannon et al. (2007) demonstrated that between 6 and 10 months of age, infants’ temporal discrimination increases in precision, such that by 10 months they can successfully discriminate a 2:3 ratio change in tempo whereas at 6 months infants require a 1:2 ratio to detect a change. Importantly, identical patterns of discrimination were also shown within infants’ numerical discrimination abilities (Xu and Spelke, 2000; Lipton and Spelke, 2003). Moreover, changes in numerical magnitude have been shown to influence adult humans’ perception of time (Xuan et al., 2007; Oliveri et al., 2008; Vicario et al., 2008; Lu et al., 2009; Chang et al., 2011; Vicario, 2011). For instance, Chang et al. (2011) demonstrated that task-irrelevant numerical magnitude significantly influenced participants’ temporal reproduction. That is, when asked to provide a key press to reproduce the duration of an Arabic numeral on a computer screen, reproductions were shorter for small numbers than for large numbers (Chang et al., 2011). These findings suggest that a common magnitude representation may underlie both numerical and temporal processing.

Finally, neuroimaging and neuropsychological studies in humans provide additional evidence that multiple magnitudes such as interval timing and enumeration recruit at least some similar neural substrates in parietal cortex (e.g., Rao et al., 2001; Pinel et al., 2004; Buhusi and Meck, 2005; Cantlon et al., 2006; Hubbard et al., 2008). While temporal estimations employ broadly distributed neural circuitry, these estimations still recruit similar regions of the posterior parietal cortex as numerical comparisons, suggesting that this parietal activation may reflect attentional processes or general quantity judgments (e.g., Rao et al., 2001; Buhusi and Meck, 2005). Conversely, activation found in corticostriatal and corticocerebellar regions may be more heavily implicating in timing-specific processes (Schubotz et al., 2000; Matell and Meck, 2004; Meck et al., 2008). Although the parietal cortex may be the main locus of overlap for processing time and number, neurons that respond to both magnitudes are also found in the dorsolateral prefrontal cortex (e.g., Nieder et al., 2002; Genovesio et al., 2006).

### CURRENT RESEARCH

Taken together, there is some support that temporal and numerical information may be simultaneously encoded and share a similar cognitive representation. Furthermore, an increasing body of research has provided evidence that emotional valence leads to an overestimation of time, while more recent findings have suggested that emotional valence results in an underestimation of numerosity (Rodzon et al., 2011; Young and Cordes, 2013). Therefore, while a relationship between the processing of time and number exists, it remains unknown whether both are processed by a shared or distinct cognitive mechanism. Thus, a significant gap remains in the literature concerning the possible influence of emotion on other quantitative properties, including numerical estimations. The aim of the present experiment is to investigate the impact of emotion on enumeration using a numerical bisection task and to provide results that may help elucidate the cognitive mechanisms that underlie time and number processing. Given the documented effects of emotion on estimations of duration, we aimed to explore whether similar effects on estimation of numerosity could be identified.

In the numerical bisection paradigm, participants are trained to discriminate between a small numerical quantity and a large numerical quantity and are then tested with intermediate numerical quantities. The intermediate quantity probe trials require participants to judge whether the probe numerosity is more similar to the small anchor quantity or the large anchor quantity. The test quantity for which participants are equally likely to choose the small or large anchor quantities, the PSE, is calculated (e.g., Droit-Volet et al., 2003; Jordan and Brannon, 2006a,b). In the current experiment, faces with positive, neutral, and negative affect were used as stimuli to enumerate. PSE’s were compared across these different stimuli types to determine whether subjective numerical perception is similar across these emotional valences.

## MATERIALS AND METHODS

### PARTICIPANTS

Participants (*n* = 33) consisted of undergraduate students enrolled in a general psychology class at Utah State University. Participants received course credit.

### MATERIALS: APPARATUS AND STIMULI

Participants were individually tested for approximately 30 min in a laboratory room. Testing consisted of a computer-based numerical bisection task that presented visual facial stimuli on a Dell Optiplex 755 computer with a 21-inch monitor. All participants sat approximately 45 cm from the computer display, and all responses were made using a keyboard. E-Prime^®^ stimulus presentation software (Schneider et al., 2002a,b) was used to present all stimuli and record responses.

**FIGURE 1:**
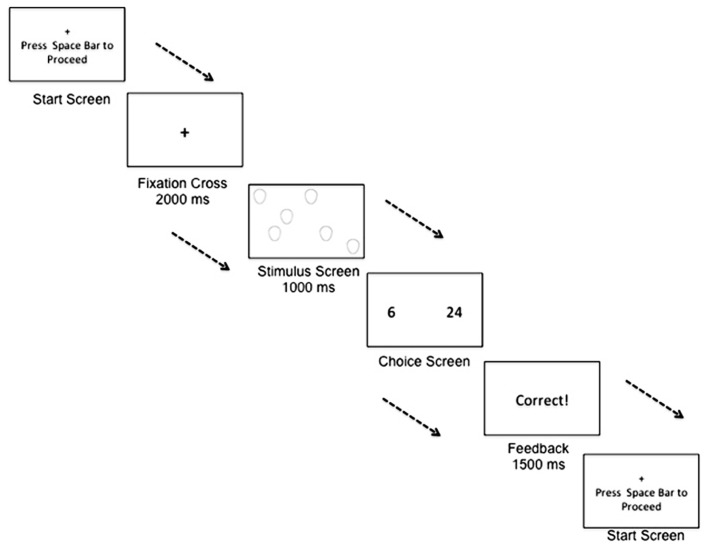
**Example of a trial within the numerical bisection training task.** A participant begins a trial by fixating on a cross. The participant is then shown a stimulus screen, displaying a number of stimuli to rapidly enumerate. The participant is then shown a choice screen, and must decide whether the stimulus numerosity was closer in numerical quantity to the small anchor quantity or the large anchor quantity.

The computer-based numerical bisection task contained two phases: (1) training phase and (2) testing phase. The arrays of stimuli during the training phase were composed of ovals; this ensured that participants were not learning the task with any facial stimuli used in the subsequent testing phase. Consistent with the stimulus emotions/genders used in studies of the effects of affect on temporal perception, such as Droit-Volet et al. (2004, 2011); Droit-Volet and Meck (2007), stimuli during the testing phase were arrays of adult female faces expressing anger, neutrality, or happiness. All stimuli were pictures of the same female individual. In addition, only one picture was used for each emotion condition so that in an “angry” stimulus of numerosity 15, for example, all 15 angry pictures in the stimulus array were the same picture repeated 15 times. Stimuli were full color pictures cropped from the shoulders up. Stimulus affect was the same within each individual face comprising a trial array. Valence and purity of expression for the facial stimulus set used were previously coded using the Facial Affect Coding System (Tracy et al., 2009). So that numerosity did not always co-vary with other quantitative properties (e.g., surface area), three face stimulus sizes were used: small (8 cm × 11 cm), medium (22 cm × 16 cm), and large (44 cm × 32 cm; Tracy et al., 2009). A repeated-measure ANOVA supported that proportion of “large” responding was not driven by congruence or incongruence of the total stimulus surface area [*F*(2,72) = 0.574, *p* = 0.566]. To vary the spatial layout of stimuli across trials, face stimulus presentation was randomized on an invisible 12 × 12 grid. Finally, as shown in **Figure 1**, all images were displayed simultaneously so that in a trial of numerosity 15, for example, all 15 images were presented at the same time and remained present for 1000 ms. In this way, the total duration of the stimulus presentation did not covary with the magnitude of the numerical presentation.

### PROCEDURE

The task took approximately 30 min to complete. During the task, participants were instructed to sit comfortably in front of the keyboard and to use two hands when responding. Pressing the space bar initiated each trials which began with 2000 ms presentation of a fixation cross before automatically proceeding to the procedure described below. For both the training and testing phases, the range for the numerical bisection task was 6–24 stimuli, with the “small” anchor quantity defined as six and the “large” anchor quantity defined as 24. This ratio between the anchor quantities is identical to the ratio used in previous duration bisection tasks investigating the effects of emotion on temporal perception (e.g., Droit-Volet et al., 2004; Droit-Volet and Meck, 2007).

During the training phase, an array of neutrally valenced ovals was presented for 1000 ms in sets of either 6 or 24 (**Figure 1**). To respond, participants were instructed to press the “d” key if the numerosity was closer to the “small” anchor or the “k” key if the numerosity was closer to the “large” anchor. Twenty-four trials, 12 for each quantity, were presented during training, and feedback was given directly following each training trial. A correct response was followed by a 1500 ms visual display of “Correct!,” while an incorrect response was followed by a 1500 ms visual display of “Incorrect.” Participants were then instructed to press the spacebar to begin the next trial.

In the testing phase, feedback was eliminated and participants were instructed to perform the same task as in training. The stimuli in the testing phase were arrays of angry, happy, and neutral facial stimuli previously described. Durations were the same as practice. During test, stimulus numerosity was randomly presented in all whole numerosity quantities between 6 and 24. Identical to the practice phase, participants were instructed to press the “d” key if the numerosity was closer to the “small” anchor or the “k” key if the numerosity was closer to the “large” anchor. There were a total of 228 trials, with each numerosity presented four times for each emotional valence (i.e., angry, neutral, and happy). Participants completed the task without taking any breaks.

## RESULTS

All participants performed at or above 90% correct during training, thereby demonstrating an understanding of the visual numerical bisection task procedure. To evaluate numerical estimation of emotional stimuli, all subsequent analyses were performed on responses recorded during test.

**FIGURE 2:**
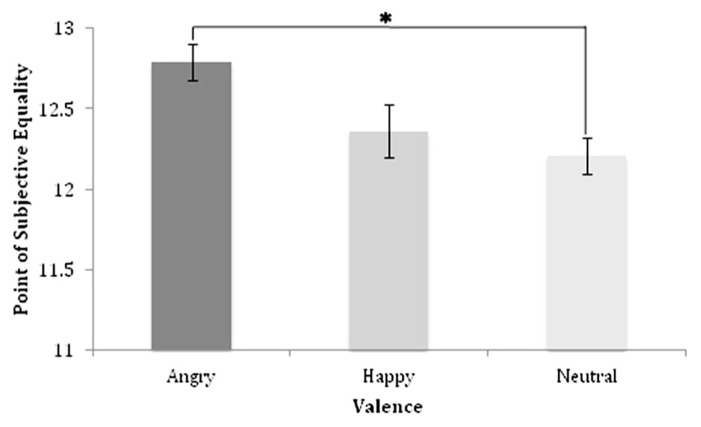
**Point of subjective equality and standard error values for each valence**.

Linear regression analysis indicated that, across all stimulus valences, proportion of “large” responses increased as stimulus numerosity increased (**Figure 2**; angry = *F*(7,32) = 15.28, MSE = 9.27, *p *< 0.001, *R*^2^ = 0.81; happy = *F*(5,32) = 8.76, MSE = 10.61, *p *< 0.001, *R*^2^ = 0.62; neutral = *F*(6,32) = 15.2, MSE = 16.55, *p *< 0.001, *R*^2^ = 0.78). These results suggest that participants showed systematic estimation of numerosity for all stimulus valences tested. Specifically, as sample numerosities increased, the tendency to judge the sample as more similar in numerosity to the large anchor numerosity also increased.

### POINT OF SUBJECTIVE EQUALITY AND SENSITIVITY TO CHANGE IN NUMBER

The PSE or the subjective midpoint between small and large anchor quantities, for each valence type was calculated based on the slope and intercept resultant from regressions of the probability of large responses at each numerosity (**Figure 2**). The PSE represents the point at which a participant is equally likely to judge a quantity as large or small, and illustrates the stimulus numerosity needed for a participant to judge a numerosity as equally close to the “small” anchor and the “large” anchor. As the arithmetic mean between each anchor quantity represents the absolute midpoint in this task, participant accuracy may be ascertained by measuring the difference between the arithmetic mean and PSE’s.

To evaluate the likelihood of any observed differences in PSE values across stimulus valence arising by chance, repeated measures analyses of variance was performed with stimulus valence as the within-subject variable. The results of this analysis revealed a significant main effect of valence in regards to PSEs [*F*(1,32) = 3.29, *p* = 0.04, Partial *η*^2^ = 0.13], indicating that valence significantly affects the numerical quantity at which a stimulus is judged to be equally distant from either anchor quantity (see **Figure 2**). Follow-up Bonferroni corrected post hoc comparisons demonstrated significantly larger PSEs for enumeration of angry facial stimuli ($\overset{-}{x}$ = 12.79, se = 0.12) compared to enumeration of neutral stimuli ($\overset{-}{x}$ = 12.20, se = 0.11; *t*(32) = 2.22, *p* = 0.03, Cohen’s *d* = 0.56), indicating that a significantly greater numerosity of angry faces compared to neutral faces were needed for participants to judge the numerical quantity to be equally distant from each endpoint. Conversely, no significant difference in PSEs was identified between happy ($\overset{-}{x}$ = 12.36, se = 0.14) and neutral facial stimuli (*p* = 0.54, Cohen’s *d* = 0.29), or between angry and happy facial stimuli (*p* = 0.07, Cohen’s *d* = 0.35). Together, these results suggest that angry facial stimuli have the largest effect on numerosity similarity judgments, which manifest in significantly larger PSE’s when enumerating angry stimuli compared to neutral stimuli.

## DISCUSSION

The aim of the current experiment was to investigate the impact of emotional valence on visual numerical similarity judgments using a numerical bisection task. To that end, we compared participants’ subjective numerical midpoints (i.e., PSE) for angry, happy, and neutral faces using repeated measures ANOVA and Bonferroni corrected *t*-tests. We observed a systematic increase in subjective numerical midpoints when the estimated stimuli were valenced. In particular, participants responded larger more often for angry compared to neutral faces. Thus, as hypothesized, valence does significantly affect numerical estimations. Specifically, direct comparisons of PSE’s resulting from estimations of each stimulus valence indicated that PSE’s for enumerating angry faces differ significantly from PSE’s for enumerating neutral faces, but only trend towards being different from PSE’s for enumerating happy faces. Thus, these results provide evidence that negative valence exerts a large influence on humans’ representation of number.

Taken together with previous research that demonstrates similar influences of negative valence on temporal estimations (Droit-Volet et al., 2004; Tipples, 2008; Gil and Droit-Volet, 2011, 2012), our results suggest that a common cognitive mechanism may underlie estimations of continuous quantities such as time and numerosity. Furthermore, our results corroborate many of the findings within the timing literature (Droit-Volet et al., 2004; Tipples, 2008; Gil and Droit-Volet, 2011; Cocenas-Silva et al., 2012), which suggest that negative valence exerts a particularly large influence on continuous quantity estimations. For example, Tipples (2008) found that the duration of angry faces was more greatly overestimated than the duration of happy faces. However, despite these similarities in the large effects of negative valences on quantitative representations, our results also reveal a peculiar disparity in regards to the direction of the PSE shift across these two quantitative domains of time and numerosity. That is, the larger PSE’s identified here for angry compared to neutral stimuli indicate that angry stimuli must be perceived as being numerically *lesser* than neutral stimuli, so that a greater number of angry faces are needed to reach the PSE. In the timing studies reported above (Droit-Volet et al., 2004, 2011; Droit-Volet and Meck, 2007; Tipples, 2008), durations of angry stimuli were overestimated compared to durations of neutral stimuli, which occurs because negatively valenced stimuli are perceived as lasting longer (i.e., temporally *greater*) than neutral stimuli. Therefore, while negative valence affects both numerical and temporal estimations, this effect seems to be in opposite directions (i.e., subjective psychophysical decrease in quantity for numerosity, but increase for time, compared to neutrally valenced stimuli). Furthermore, in the current study, the number of angry faces was underestimated to a greater extent than the number of happy faces (though both types of valenced faces were underestimated more than neutral faces). Young and Cordes (2013), in contrast, found that the number of happy faces was underestimated more than the number of angry faces. This difference could be due to slight experimental design differences between the two experiments – e.g., different ratios of anchor values used, different numbers of intermediate probe values used, or participants in Young and Cordes’ study also completing a temporal bisection task in the same session – and remains to be directly tested in the future.

This directional disparity may be indicative of related but distinct neurological processes that underlie perception of time and number, although both properties likely rely on scalar internal representation (Wearden, 1991; Wearden and Ferrara, 1996). That is, the fact that perception of both quantitative domains is affected by emotional valence suggests a common mechanism, although the opposite directionality of such effects suggests the possibility of different underlying processes. For example, Droit-Volet et al. (2004) have argued that an arousal-modulated process underlies temporal estimation, such that increased arousal results in an overestimation of perceived duration. Alternatively, Young and Cordes (2013) have argued that numerical estimations may be influenced by attentional focusing, resulting in heightened attention to arousing stimuli and coincident failure to encode each enumerable element within a visual display, thus resulting in numerical underestimation. Importantly, similar attention-based claims have been made for cognitive abilities in response to emotional stimuli in domains including visual search (Öhman and Esteves, 2001; Öhman et al., 2001), memory (MacKay and Ahmetzanov, 2005), Stroop color-naming (Siegrist, 1995; Williams et al., 1996), and others. Future studies should thus aim to tease apart potential effects of arousal vs. attention in both temporal and numerical estimation tasks, to better determine whether data truly support a common system of generalized magnitude processing. If future research shows further evidence that attention and arousal exert separate effects on our processing of time and number, this may support multiple systems of magnitude processing instead.

Interestingly, when viewed through the lens of proximity to the arithmetic or geometric mean between the numerical anchors used in the current study (6 vs. 24), our results suggest that angry valenced stimuli may enhance our approximate numerical representations. As reviewed in the Section “Introduction,” under conditions in which no source of emotion is present, humans’ representations of numerosity are approximate, and discrimination conforms to Weber’s Law with “noisy” scalar distributions situated around the geometric midpoint between competing stimuli (Gallistel and Gelman, 2000). In the current experiment, the tendency for PSE’s from numerical estimations of angry valenced stimuli to deviate away from the geometric midpoint towards the arithmetic midpoint may be a result of more accurate (i.e., less “noisy”) numerosity representations (see Jordan and Brannon, 2006a,b, for further comparisons of PSE’s in numerical bisection tasks to arithmetic vs. geometric means). It is possible that negatively valenced stimuli recruit greater attentional resources compared with neutral stimuli, thereby resulting in PSE’s that are closer to the more accurate arithmetic midpoint. In this regard, negatively valenced stimuli may be acting upon humans’ numerosity perceptions in a way that is similar to the effect of intersensory redundancy, which has also been shown to enhance psychophysical discrimination of quantitative stimuli including numerosity (e.g., Jordan et al., 2008a,b; Jordan and Baker, 2011) and time (Bahrick and Lickliter, 2000). However, as PSEs have been shown to vary widely across disparate applications of the bisection task, with some groups reporting PSEs closer to the arithmetic mean (e.g., Droit-Volet et al., 2004; Balci and Gallistel, 2006) and others reporting PSEs closer to the geometric mean (e.g., Wearden and Ferrara, 1996; Wearden et al., 1997; Zélanti and Droit-Volet, 2011), a benchmark of optimal bisection performance remains under investigation. If bisection at the geometric mean were indeed considered accurate responding in accord with some of these studies, then emotion may actually decrease accuracy of numerical responding. Caution is thus warranted in drawing strong conclusions about accuracy from this bisection task.

The generalization of these findings to numerical judgments suggests that related effects may also occur in subjective perception of other types of quantities other than number and time, such as discriminations of length, surface area, volume, and others. As described above, it has been hypothesized that similar mental algorithms and neural areas may be devoted to magnitude processing in general (e.g., Walsh, 2003; Cantlon et al., 2006). Our results support this claim, while also suggesting that this common mechanism may be compartmentalized to some degree so that certain subsections are devoted to specific properties and may thus respond slightly differently to the presence of negative valence. It will be important to determine the extent to which emotion also colors our perception of other quantitative properties, and furthermore, to identify neural circuitry involved in such processing. Studies investigating the effects of emotion on processing of other magnitudes – such as spatial extent – and other modalities are thus needed. Together, this body of research will elucidate whether a similar, modality-specific or modality-independent mechanism may affect processing of magnitude, or whether distinct mechanisms underlie processing of each magnitude type. An understanding of how emotion alters our quantitative processing could also inform our understanding of relevant dysfunction in those with a mood disorder (e.g., temporal dysfunction in anxiety).

The current study sets the stage for much future research. For example, although previous research using similar emotional stimuli as in the current study found consistent effects of such emotional stimuli across entire sessions of timing tasks (e.g., Droit-Volet et al., 2004, 2011; Gil and Droit-Volet, 2011), we did not directly measure emotional state of participants across the testing session here. It would be useful in the future to measure subjective feelings of emotion experienced by the participants when viewing these emotional pictures across the testing session. Furthermore, additional research is required in order to fully understand if emotion impacts visual numerical representations similarly across valence. Finally, the effects of emotion on enumeration of auditory stimuli remains unknown. Time and numerosity are amodal properties, and emotion has been shown to impact timing (e.g., Angrilli et al., 1997; Noulhiane et al., 2007; Mella et al., 2011). Kanai and Rees (2011) even show that auditory cortex may prove superior in some aspects of temporal processing; thus, there may similarly remain modalities other than vision that are more sensitive to the influence of affect when processing numerosity as well.

## Conflict of Interest Statement

The authors declare that the research was conducted in the absence of any commercial or financial relationships that could be construed as a potential conflict of interest.

## References

[B1] Allman M. J. Pelphrey K. A. Meck W. H. (2012). Developmental neuroscience of time and number: implications for autism and other neurodevelopmental disorders. *Front. Integr. Neurosci. * 6:7 10.3389/fnint.2012.00007 PMC3294544 22408612

[B2] Angrilli A. Cherubini P. Pavese A. Manfredini S. (1997). The influence of affective factors on time perception. *Percept. Psychophys.* 59 972–982 10.3758/BF03205512 9270369

[B3] Bahrick L. E. Lickliter R. (2000). Intersensory redundancy guides attentional selectivity and perceptual learning in infancy. * Dev. Psychol.* 36 190–201 10.1037//0012-1649.36.2.190 10749076 PMC2704001

[B4] Baker J. M. Morath J. Rodzon K. R. Jordan K. E. (2012). A shared system of representation governing quantity discrimination in canids. *Front. Psychol.* 3:387 10.3389/fpsyg.2012.00387 PMC3465982 23060847

[B5] Baker J. M. Shivik J. Jordan K. E. (2011). Tracking of food quantity by coyotes (*Canis latrans*). *Behav. Processes* 88 72–75 10.1016/j.beproc.2011.08.006 21856389

[B6] Balci F. Gallistel C. R. (2006). Cross-domain transfer of quantitative discriminations: is it all a matter of proportion? *Psychon. Bull. Rev*. 13 636–642 10.3758/BF03193974 17201363

[B7] Brannon E. M. Roitman J. D. (2003). “Nonverbal representations of time and number in animals and human infants,” in *Functional and Neural Mechanisms of Interval Timing* ed. Meck W. H. (UK: CRC Press) 143–182

[B8] Brannon E. M. Suanda S. Libertus K. (2007). Temporal discrimination increases in precision over development and parallels the development of numerosity discrimination. *Dev. Sci.* 10 770–777 10.1111/j.1467-7687.2007.00635.x 17973794 PMC2918408

[B9] Brown S. W. (1997). Attentional resources in timing: interference effects in concurrent temporal and non-temporal working memory tasks. *Percept. Psychophys*. 59 1118–1140 10.3758/BF03205526 9360484

[B10] Bueti D. Walsh V. (2009). The parietal cortex and the representation of time, space, number and other magnitudes. *Philos. Trans. R. Soc. B Biol. Sci.* 364 1831–1840 10.1098/rstb.2009.0028 PMC2685826 19487186

[B11] Buhusi C. V. Meck W. H. (2005). What makes us tick? Functional and neural mechanisms of interval timing. *Nat. Rev. Neurosci.* 6 755–765 10.1038/nrn1764 16163383

[B12] Cantlon J. F. Brannon E. M. Carter E. J. Pelphrey K. A. (2006). Functional imaging of numerical processing in adults and 4-y-old children. *PLoS Biol.* 4:e125 10.1371/journal.pbio.0040125 PMC1431577 16594732

[B13] Chang A. Y. C. Tzeng O. J. L. Hung D. L. Wu D. H. (2011). Big time is not always long: numerical magnitude automatically affects time reproduction. *Psychol. Sci.* 22 1567–1573 10.1177/0956797611418837 22042728

[B14] Cocenas-Silva R. Bueno J. L. O. Droit-Volet S. (2012). Temporal memory of emotional experience. *Mem. Cognit.* 40 161–167 10.3758/s13421-011-0145-5 21948305

[B15] Cohen Kadosh R. Lammertyn J. Izard V. (2008). Are numbers special? An overview of chronometric, neuroimaging, developmental and comparative studies of magnitude representation. *Prog. Neurobiol.* 84 132–147 10.1016/j.pneurobio.2007.11.001 18155348

[B16] Dehaene S. Dehaene-Lambertz G. Cohen L. (1998). Abstract representations of number in the animal and human brain. *Trends Neurosci.* 21 355–366 10.1016/S0166-2236(98)01263-6 9720604

[B17] Dormal V. Andres M. Pesenti M. (2008). Dissociation of numerosity and duration processing in the left intraparietal sulcus: a transcranial magnetic stimulation study. *Cortex* 44 462–469 10.1016/j.cortex.2007.08.011 18387579

[B18] Dormal V. Grade S. Mormont E. Pesenti M. (2012). Dissociation between numerosity and duration processing in aging and early Parkinson’s disease. *Neuropsychologia* 50 2365–2370 10.1016/j.neuropsychologia.2012.06.006 22728343

[B19] Droit-Volet S. Brunot S. Niedenthal P. M. (2004). Perception of duration of emotional events. *Cogn. Emot.* 18 849–858 10.1080/02699930341000194

[B20] Droit-Volet S. Clement A. Fayol M. (2003). Time and number discrimination in a bisection task with a sequence of stimuli: a developmental approach. *J. Exp. Child Psychol.* 84 63–76 10.1016/S0022-0965(02)00180-7 12553918

[B21] Droit-Volet S. Fayollee S. L. Gil S. (2011). Emotion and time perception: effects of film-induced mood. *Front. Integr. Neurosci.* 5:1–9 10.3389/fnint.2011.00033 21886610 PMC3152725

[B22] Droit-Volet S. Meck W. H. (2007). How emotions color our perception of time. *Trends Cogn. Sci.* 11 504–513 10.1016/j.tics.2007.09.008 18023604

[B23] Fetterman J. G. (1993). Numerosity discrimination: both time and number matter. *J. Exp. Psychol. Anim. Behav. Process.* 19 149–164 10.1037/0097-7403.19.2.149 8505595

[B24] Gallistel C. R. Gelman R. (2000). Non-verbal numerical cognition: from reals to integers. *Trends Cogn. Sci.* 4 59–65 10.1016/S1364-6613(99)01424-2 10652523

[B25] Genovesio A. Tsujimoto S. Wise S. P. (2006). Neuronal activity related to elapsed time in prefrontal cortex *J. Neurophysiol.* 95 3281–3285 10.1152/jn.01011.2005 16421197 PMC1475947

[B26] Gil S. Droit-Volet S. (2011). “Time flies in the presence of angry faces”⋯depending on the temporal task used! Acta Psychol. 136 354–362 10.1016/j.actpsy.2010.12.010 21276583

[B27] Gil S Droit-Volet S. (2012). Emotional time distortions: the fundamental role of arousal. *Cogn. Emot.* 26 847–862 10.1080/02699931.2011.625401 22296278

[B28] Gil S. Niedenthal P. M. Droit-Volet S. (2007). Anger and time perception in children. *Emotion* 7 219–225 10.1037/1528-3542.7.1.219 17352578

[B29] Hubbard E. M. Diester I. Cantlon J. F. Ansari D. van Opstal F. Troiani V. (2008). The evolution of numerical cognition: from number neurons to linguistic quantifiers. *J. Neurosci.* 28 11819–11824 10.1523/JNEUROSCI.3808-08.2008 19005046 PMC2709405

[B30] Jordan K. E. Baker J. (2011). Multisensory information boosts numerical matching abilities in young children. *Dev. Sci.* 14 205–213 10.1111/j.1467-7687.2010.00966.x 22213895

[B31] Jordan K. E. Brannon E. M. (2006a). A common representational system governed by Weber’s law: nonverbal numerical similarity judgments in 6-year-olds and rhesus *macaques*. *J. Exp. Child Psychol.* 95 215–229 10.1016/j.jecp.2006.05.004 16808924

[B32] Jordan K. E. Brannon E. M. (2006b). Weber’s law influences numerical representations in rhesus *macaques* (*Macaca mulatta*). *Anim. Cogn.* 9 159–172 10.1007/s10071-006-0017-8 16575587

[B33] Jordan K. E. MacLean E. L. Brannon E. M. (2008a). Monkeys match and tally quantities across senses. *Cognition* 108 617–625 10.1016/j.cognition.2008.05.006 18571636 PMC3641156

[B34] Jordan K. E. Suanda S. H. Brannon E. M. (2008b). Intersensory redundancy accelerates preverbal numerical competence. *Cognition* 108 210–221 10.1016/j.cognition.2007.12.001 18226807 PMC2768652

[B35] Kanai R. Rees G. (2011). The structural basis of inter-individual differences in human behavior and cognition. *Nat. Rev. Neurosci.* 12 231–242 10.1038/nrn3000 21407245

[B36] Lipton J. S. Spelke E. S. (2003). Origins of number sense: large-number discrimination in human infants. *Psychol. Sci.* 14 396–401 10.1111/1467-9280.01453 12930467

[B37] Lourenco S. F. Longo M. R. (2010). General magnitude representation in human infants. *Psychol. Sci.* 21 873–881 10.1177/0956797610370158 20431048 PMC2930776

[B38] Lu A. Hodges B. Zhang J. Zhang J. X. (2009). Contextual effects on number-time interaction. *Cognition* 113 117–122 10.1016/j.cognition.2009.07.001 19640515

[B39] MacKay D. G. Ahmetzanov M. V. (2005). Emotion, memory, and attention in the taboo Stroop paradigm. *Psychol. Sci.* 16 25–32 10.1111/j.0956-7976.2005.00776.x 15660848

[B40] Matell M. S. Meck W. H. (2004). Cortico-striatal and interval timing: coincidence detection of oscillatory processes. *Cogn. Brain Res.* 21 139–170 10.1016/j.cogbrainres.2004.06.012 15464348

[B41] Meck W. H. (1983). Selective adjustment of the speed of internal clock and memory processes. *J. Exp. Psychol. Anim. Behav. Process.* 120 1163–1168 10.1037/0097-7403.9.2.171 6842136

[B42] Meck W. H. Church R. M. (1983). A mode control model of counting and timing processes. *J. Exp. Psychol. Anim. Behav. Process.* 9 320–334 10.1037/0097-7403.9.3.320 6886634

[B43] Meck W. H. Penney T. B. Pouthas V. (2008). Cortico-striatal representation of time in animals and humans. *Curr. Opin. Neurobiol.* 18 145–152 10.1016/j.conb.2008.08.002 18708142

[B44] Mella N. Conty L. Pouthas V. (2011). The role of physiological arousal in time perception: psychophysiological evidence from an emotion regulation paradigm. *Brain Cogn.* 75 182–187 10.1016/j.bandc.2010.11.012 21145643

[B45] Moyer R. S. Landauer T. K. (1967). Time required for judgments of numerical inequality. *Nature* 215 1519–1520 10.1038/2151519a0 6052760

[B46] Nieder A. Freedman D. J. Miller E. K. (2002). Representation of the quantitiy of visual items in the primate prefrontal cortex. *Science* 297 1708–1711 10.1126/science.1072493 12215649

[B47] Noulhiane M. Mella N. Samson S. Ragot R. Pouthas V. (2007). How emotional auditory stimuli modulate time perception. *Emotion* 7 697–704 10.1037/1528-3542.7.4.697 18039036

[B48] öhman A. Esteves F. (2001). Emotion drives attention: detecting the snake in the grass. *J. Exp. Psychol.* 130 466–478 10.1037/0096-3445.130.3.466 11561921

[B49] öhman A. Lundqvist D. Esteves F. (2001). The face in the crowd revisited: a threat advantage with schematic stimuli. *J. Pers. Soc. Psychol.* 80 381–396 10.1037/0022-3514.80.3.381 11300573

[B50] Oliveri M. Vicario C. M. Salerno S. Koch G. Turriziani P. Mangano R. (2008). Perceiving numbers alters time perception. *Neurosci. Lett.* 438 308–311 10.1016/j.neulet.2008.04.051 18486340

[B51] Pinel P. Piazza M. Le Bihan D. Dehaene S. (2004). Distributed and overlapping cerebral representations of number, size, and luminance during comparative judgements. *Neuron* 41 983–993 10.1016/S0896-6273(04)00107-2 15046729

[B52] Rao S. M. Mayer A. R. Harrington D. (2001). The evolution of brain activation during temporal processing. *Nat. Neurosci.* 4 317–323 10.1038/85191 11224550

[B53] Roberts W. A. Coughlin R. Roberts S. (2000). Pigeons flexibility time or count on cue. *Psychol. Sci.* 11 218–222 10.1111/1467-9280.00244 11273406

[B54] Rodzon K. S. Baker J. M. Jordan K. E. (2011). “The impact of emotion on numerical estimation,” in *Proceedings of the 32nd Annual Conference of the Cognitive Science Society* Boston, MA 3552–3557

[B55] Roitman J. D. Brannon E. M. Platt M. L. (2007). Monotonic coding of numerosity in *macaque* lateral intraparietal area. *PLoS Biol.* 5:e208 10.1371/journal.pbio.0050208 PMC1925133 17676978

[B56] Schiff W. Thayer S. (1970). Cognitive and affective factors in temporal experience: judgment of intrinsically motivated successful and unsuccessful performances. *Percept. Mot. Skills* 30 895–902 10.2466/pms.1970.30.3.895

[B57] Schneider W. Eschman A. Zuccolotto A. (2002a). *E-Prime User’s Guide*. Pittsburgh: Psychology Software Tools, Inc

[B58] Schneider W. Eschman A. Zuccolotto A. (2002b). *E-Prime Reference Guide*. Pittsburgh: Psychology Software Tools, Inc

[B59] Schubotz R. I. Friederici A. D Yves von Cramon D. (2000). Time perception and motor timing: a common cortical and subcortical basis revealed by fMRI. *NeuroImage* 11 1–12 10.1006/nimg.1999.0514 10686112

[B60] Siegrist M. (1995). Effects of taboo words on color-naming performance on a Stroop test. *Percept. Mot. Skills* 81 1119–1122 10.2466/pms.1995.81.3f.1119 8684902

[B61] Stetson C. Fiesta M. P. Eagleman D. M. (2007). Dies time really slow down during a frightening event? *PLoS ONE* 2:e1295 10.1371/journal.pone.0001295 PMC2110887 18074019

[B62] Stevens S. S. (1957). On the psychophysical law. *Psychol. Rev.* 64 153–181 10.1037/h0046162 13441853

[B63] Tipples J. (2008). Negative emotionality influences the effects of emotion on time perception. *Emotion* 8 127–131 10.1037/1528-3542.8.1.127 18266523

[B64] Tomkins S. S. Karon B. P. (eds). (1962) Affect Imagery Consciousness: The Complete Edition. New York: Springer PublishingCompany, LLC

[B65] Tracy J. L. Robins R. W. Schriber R. A. (2009). Development of a FACS-verified set of basic and self-conscious emotion expressions. *Emotion* 9 554–559 10.1037/a0015766 19653779

[B66] Vicario C. M. (2011). Perceiving numbers affects the subjective temporal midpoint. *Perception* 40 23–29 10.1068/p6800 21513181

[B67] Vicario C. M. Pecoraro P. Turriziani P. Koch G. Caltagirone C. Oliveri M. (2008). Relativistic compression and expansion of experiential time in the left and right space. *PLoS ONE* 3:e1716 10.1371/journal.pone.0001716 PMC2248621 18320037

[B68] Walsh V. (2003). A theory of magnitude: common cortical metrics of time, space and quantity. *Trends Cogn. Sci.* 7 483–488 10.1016/j.tics.2003.09.002 14585444

[B69] Wearden J. H. (1991). Do humans possess an internal clock with scalar timing properties? *Learn. Motiv.* 22 59–83 10.10.1016/0023-9690(91)90017-3

[B70] Wearden J. H. Ferrara A. (1996). Stimulus range effects in temporal bisection of humans. *Q. J. Exp. Psychol. B* 49 24–44 10.1080/713932615 8901385

[B71] Wearden J. H. Rogers P. Thomas R. (1997). Temporal bisection in humans with longer stimulus durations. *Q. J. Exp. Psychol. B* 50 79–94 10.1080/027249997393655 9141912

[B72] Williams J. M. G. Mathews A. MacLeod C. (1996). The emotional Stroop task and psychopathology. *Psychol. Bull.* 120 3–24 10.1037/0033-2909.120.1.3 8711015

[B73] Xu F. Spelke E. S. (2000). Large number discrimination in 6-month-old infants. *Cognition* 74 B1–B11 10.1016/S0010-0277(99)00066-9 10594312

[B74] Xuan B. Zhang D. He S. Chen X. (2007). Larger stimuli are judged to last longer. *J. Vis.* 7 1–5 10.1167/7.10.2 17997671

[B75] Young L. N. Cordes S. (2013). Fewer things, lasting longer: the effects of emotion on quantity judgements. *Psychol. Sci.* 1057–1059 10.1177/0956797612465294 23603915

[B76] Zélanti P. S. Droit-Volet S. (2011). Cognitive abilities explaining age-related changes in time perception of short and long durations. *J. Exp. Child Psychol.* 109 143–157 10.1016/j.jecp.2011.01.003 21334637

